# A case of bronchial atresia treated with right S6 segmentectomy using fluorescence navigation with indocyanine green

**DOI:** 10.1186/s44215-022-00009-y

**Published:** 2022-12-01

**Authors:** Mototsugu Watanabe, Kaoru Kondo, Shinichi Furukawa, Toshio Shiotani, Kazuhiko Kataoka

**Affiliations:** Department of Thoracic Surgery, National Hospital Organization Iwakuni Clinical Center, 1-1-1 Atago-machi, Iwakuni-shi, Yamaguchi 740-8510 Japan

**Keywords:** Bronchial atresia, Video-assisted thoracoscopic surgery (VATS), Segmentectomy, Indocyanine green (ICG)

## Abstract

**Background:**

Bronchial atresia is a congenital obstruction of the segmental or lobar bronchus that often leads to hyperinflation of the affected area. It can cause intractable infections and abnormal nodules in these regions and surgical resection needs to be considered. The precise resection of the abnormal pulmonary segment is crucial for diagnosis and treatment.

**Case presentation:**

A 24-year-old male patient was incidentally diagnosed with an abnormal lung shadow, which was confirmed to be bronchial atresia with computerized tomography imaging and bronchofiberscopy. Segmentectomy using fluorescence navigation with indocyanine green dye was performed. The patient had no trouble in his clinical course, indicating that this procedure may be useful in the resection of congenital bronchial atresia regions.

**Conclusion:**

Segmentectomy using indocyanine green is an appropriate technique for minimal resection without respiratory impairment and diagnostic therapy of bronchial atresia.

## Background

Bronchial atresia is a congenital abnormality of the tracheobronchial tree and is often identified as an incidental finding in routine examinations [1].

The actual cause of bronchial atresia is unclear. As normal bronchial patterns are often present distally to the site of stenosis, it has been suggested that the occlusion is probably not a result of abnormal growth and development but rather secondary to a traumatic event during fetal life [2]. The most sensitive imaging modality used in the diagnoses of bronchial atresia is computerized tomography (CT), with most common findings of (1) a mucocele (100%), (2) emphysematous changes caused by hyperinflation in the peripheral lung field (100%), (3) the occlusion of the bronchus central to the mucocele (26%), and (4) bronchial wall thickening (17%) [3–5]. In some cases, it may be difficult to diagnose whether an abnormal shadow is a mucocele or a neoplasm at an abnormal pulmonary segment because we cannot reach the affected area as a result of bronchial obstruction by bronchofiberscopy. In addition, the presence of emphysematous changes makes it difficult to perform a CT-guided lung biopsy. Therefore, the precise resection of the abnormal pulmonary segment is crucial for both the diagnosis and treatment.

In this report, we describe a case of bronchial atresia treated with a complete thoracoscopy-assisted right superior (S6) segmentectomy using indocyanine green (ICG) dye imaging.

## Case presentation

**Fig. 1:**
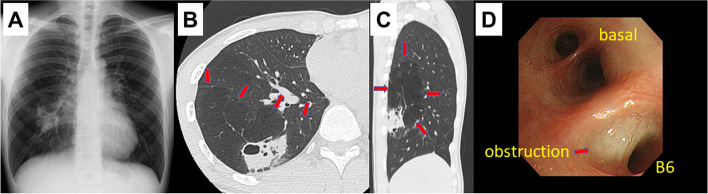
**A** Initial chest radiograph showed an abnormal shadow in the right lower lung field. **B**, **C** Chest CT scan revealed an abnormal mass with an emphysematous change. Arrows show the emphysematous region. **D** Obstruction of the bronchi was found by bronchofiberscopy

A 24-year-old male patient presented with an area of increased opacity on radiography in his annual medical examination (Fig. 1A) and was referred to our hospital for further examination and treatment.

The patient was a current smoker (3-pack-year history) but did not have previous medical or family history. A contrast-enhanced CT scan revealed an abnormal shadow in the superior segment of the right lower lobe and an emphysematous change around the affected area (Fig. 1B, C). Furthermore, CT imaging showed a suspicion of bronchial atresia, and no blood vessel abnormalities, which excluded the differential diagnosis of intralobar pulmonary sequestration. The bronchofiberscopy revealed an abnormal bronchial occlusion near the B6 segment (Fig. 1D). The laboratory data analysis showed that tumor markers and fungal antigens were not elevated. Finally, we determined that the best choice would be to perform a surgical resection of the abnormal emphysematous segment and nodular mass, as it served as the diagnostic therapy option.

**Fig. 2:**
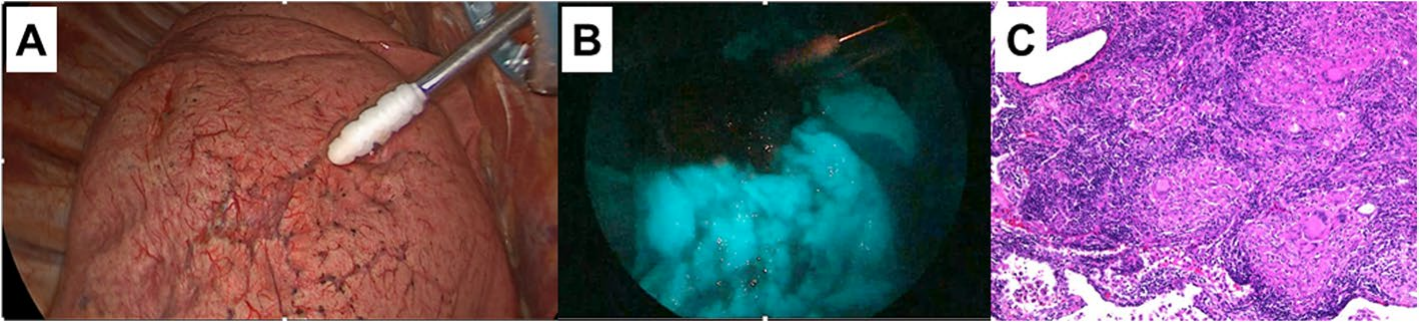
**A** Thoracoscopic view at the beginning of the operation. **B** Fluorescence navigation with ICG clearly showed the intersegmental plane. **C** The pathological report showed a granuloma without any malignant cells (magnification ×100)

The operation performed was a video-assisted thoracoscopic surgery (VATS), with the placement of the port site with a 3-cm working incision at the sixth intercostal space in the posterior axillary line, a 1-cm incision at the fifth intercostal space in the posterior axillary line, a 2-cm incision at the fourth intercostal space in the anterior axillary line, and a 2-cm incision at the seventh intercostal space in the midaxillary line. There were no specific findings on the surface of the right lower lobe during the procedure (Fig. 2A). We performed ligation and stapling for the corresponding superior segmental pulmonary artery, vein, and bronchus. The blood vessels were a little atrophic, but not particularly fragile. The bronchi could also be resected at the root of B6, and no abnormalities were found in the surgical findings. Then, an injection of ICG (12.5 mg/body) was rapidly infused through a peripheral vein. Tissues with existent blood flow were infused with the green dye 30–40 s after the ICG injection, while the isolated segment remained uncolored (Fig. 2B). Using that color change as a landmark, segmental lineage was performed by stapling, which precipitated the superior segmentectomy of the right lower lobe. The postoperative course was uneventful. The removed tumor was pathologically diagnosed as a granuloma, which was not the cause of the obstruction (Fig. 2C). The follow-up chest CT scan 6 months after the surgery showed no new or leftover emphysematous changes, lesions, or nodules in the right lung. In the follow-up period of 3 years, there was no recurrence of bronchial atresia, emphysematous changes, or any infection in other lobes.

## Discussion and conclusions

Bronchial atresia is a congenital anomaly resulting from the focal interruption of a lobar, segmental, or subsegmental bronchus with associated peripheral mucus impaction (bronchocele, mucocele) and hyperinflation of the obstructed lung segment [1]. CT is the most sensitive imaging modality and can characterize the lack of communication between the mucocele and the pulmonary hilum [3, 4]. A typical radiographic finding of bronchial atresia is a branching tubular or nodular area of increased opacity that extends from the hilum with surrounding hyperlucent lung parenchyma [6].

Although no surgical intervention is necessary for asymptomatic patients, careful observation of individual cases is required. Bronchial atresia can often cause severe pulmonary infections that cannot be controlled by antibiotics; thus, the treatment option would be the resection of the abnormal regions [3]. In addition, abnormal nodules can appear in the affected area. It is extremely difficult to make an accurate diagnosis from an abnormal nodule or mass by bronchofiberscopy because of the lack of bronchi in a tumor. Lobectomy is the most frequently reported procedure for the treatment of a pulmonary lobe with such an abnormal region [7]. However, segmentectomy is more appropriate for diagnostic therapy because it causes minimal respiratory impairment. This method is sometimes challenging since the residual normal lung tissue is usually compressed by the emphysematous area, and the intersegmental plane changes are unclear [8]. Since congenital bronchial atresia is not a malignant disease, a thoracoscopic approach should be performed whenever possible [9].

In our case, as the patient did not have any symptoms or history of pneumonia, we could not completely exclude the possibility of a malignant tumor. However, it was difficult to obtain the correct diagnosis from the detected abnormality as bronchofiberscopy and CT-guided biopsy were not suitable because of the bronchial obstruction. In this case, B6c was found to be obstructed by bronchoscopy. In addition, emphysematous changes in the lung parenchyma affected the entire S6; therefore, it was easy to determine the extent of resection. We decided that segmentectomy was the appropriate procedure for the treatment. When applied to other cases, we believe it is possible to determine the vessels to be resected by comprehensively judging the findings of bronchoscopy and CT.

For segmentectomy operation, there are two well-known methods. One uses selective segmental inflation via bronchofiberscopy and the other uses ICG dye [10, 11]. The ICG dye was developed for near infra-red photography and was approved for clinical use in 1959 by the Food and Drug Administration [12]. ICG binds rapidly to plasma lipoproteins and becomes fluorescent when excited by light or a laser beam at specific wavelengths in the near-infra-red spectrum (approximately 820 nm) [13]. The fluorescence can be detected using specific scopes and cameras (KARL STORZ, SE & Co. KG, Tuttlingen, Germany). Under the infrared light, the lung was clearly separated into two areas, in line with the existence of the blood flow on the monitor [14]. Then, we were able to perform a clear resection of the target region. Fluorescence navigation with ICG is a useful and safe method for the detection of the intersegmental border and can facilitate anatomical segmentectomy even when bronchial atresia causes an abnormal change in the lung anatomy. Despite the fact that selective segmental inflation using jet ventilation is also typically used in lung segmentectomy [10], ICG may be more appropriate in the resection of the hyperinflated area as it is impossible to put the bronchofiberscope into the obstructed bronchus. In addition, for the identification of the intersegmental plane, ICG use showed an 88.0% rate of good results, which was better than the 78.7% rate achieved by the high flow jet ventilation [11].

Segmentectomy using ICG is an appropriate technique for minimal resection without respiratory impairment and diagnostic therapy of bronchial atresia.

## Data Availability

Not applicable.

## Abbreviations

CT: Computerized tomography
ICG: Indocyanine green
VATS: Video-assisted thoracoscopic surgery

## References

[1] Ramsay BH. Mucocele of the lung due to congenital obstruction of a segmental bronchus; a case report; relationship to congenital cystic disease of the lung and to congenital bronchiectasis. Dis Chest. 1953;24:96–103. 13060202 10.1378/chest.24.1.96

[2] Schuster SR, Harris GB, Williams A, Kirkpatrick J, Reid L. Bronchial atresia: a recognizable entity in the pediatric age group. J Pediatr Surg. 1978;13:682–9. 731369 10.1016/s0022-3468(78)80114-6

[3] Wang Y, Dai W, Sun Y, Chu X, Yang B, Zhao M. Congenital bronchial atresia: diagnosis and treatment. Int J Med Sci. 2012;9:207–12. 22408569 10.7150/ijms.3690 PMC3298011

[4] Kinsella D, Sissons G, Williams MP. The radiological imaging of bronchial atresia. Br J Radiol. 1992;65:681–5. 1393394 10.1259/0007-1285-65-776-681

[5] Puglia EBMD, Rodrigues RS, Daltro PA, Souza AS Jr, Paschoal MM, Labrunie EM, et al. Tomographic findings in bronchial atresia. Radiol Bras. 2021;54:9–14. 33574627 10.1590/0100-3984.2019.0136 PMC7863713

[6] Gipson MG, Cummings KW, Hurth KM. Bronchial Atresia. RadioGraphics. 2009;29:1531–5. 19755610 10.1148/rg.295085239

[7] Seo T, Ando H, Kaneko K, Ono Y, Tainaka T, Sumida W, et al. Two cases of prenatally diagnosed congenital lobar emphysema caused by lobar bronchial atresia. J Pediatr Surg. 2006;41:e17–20. 10.1016/j.jpedsurg.2006.08.037 17101340

[8] Cappeliez S, Lenoir S, Validire P, Gossot D. Totally endoscopic lobectomy and segmentectomy for congenital bronchial atresia. Eur J Cardiothorac Surg. 2009;36:222–4. 19372046 10.1016/j.ejcts.2009.02.051

[9] Traibi A, Seguin-Givelet A, Grigoroiu M, Brian E, Gossot D. Congenital bronchial atresia in adults: thoracoscopic resection. J Vis Surg. 2017;3:174. 29302450 10.21037/jovs.2017.10.15 PMC5730535

[10] Okada M, Mimura T, Ikegaki J, Katoh H, Itoh H, Tsubota N. A novel video-assisted anatomic segmentectomy technique Selective segmental inflation via bronchofiberoptic jet followed by cautery cutting. J Thorac Cardiovasc Surg. 2007;133:753–8. 17320579 10.1016/j.jtcvs.2006.11.005

[11] Yotsukura M, Okubo Y, Yoshida Y, Nakagawa K, Watanabe SI. Indocyanine green imaging for pulmonary segmentectomy. JTCVS techniques. 2021;6:151–8. 34318180 10.1016/j.xjtc.2020.12.005 PMC8300924

[12] Alander JT, Kaartinen I, Laakso A, Pätilä T, Spillmann T, Tuchin VV, et al. A review of indocyanine green fluorescent imaging in surgery. J Biomed Imaging. 2012;2012:940585. 10.1155/2012/940585 PMC3346977 22577366

[13] Boni L, David G, Mangano A, Dionigi G, Rausei S, Spampatti S, et al. Clinical applications of indocyanine green (ICG) enhanced fluorescence in laparoscopic surgery. Surg Endosc. 2015;29:2046–55. 25303914 10.1007/s00464-014-3895-x PMC4471386

[14] Misaki N, Chang SS, Igai H, Tarumi S, Gotoh M, Yokomise H. New clinically applicable method for visualizing adjacent lung segments using an infrared thoracoscopy system. J Thorac Cardiovasc Surg. 2010;140:752–6. 20850654 10.1016/j.jtcvs.2010.07.020

